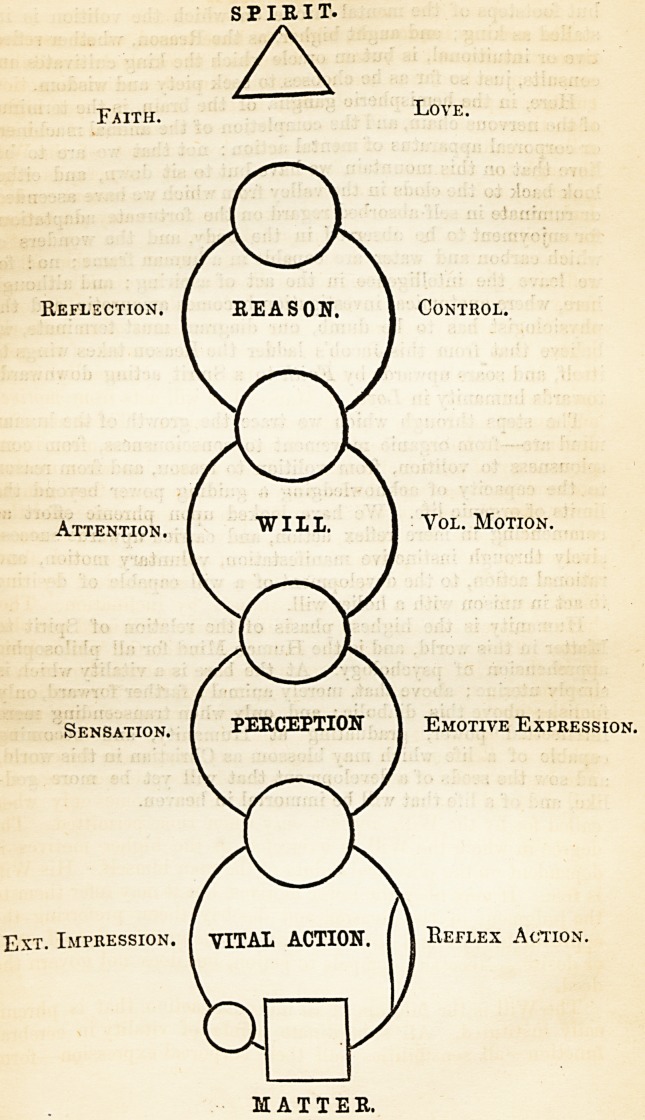# Mind and Body

**Published:** 1858-04-01

**Authors:** Robert Jamieson

**Affiliations:** President of the Medico-Chirurgical Society of Aberdeen, Medical Superintendent of the Royal Lunatic Asylum, &c.


### ART. VI.—MIND AND BODY.

A LECTURE, BY ROBERT JAMIESON, M.D.,

*President of the Medico-Chirurgical Society of Aberdeen, Medical Superintendent of the Royal Lunatic Asylum, &c.*

THE object of this paper is to illustrate the presumed course of nervous movement concerned in the mental phenomena which are associated with the Brain. In the title, the use of the term *Phrenic*, or *Phrenical*, is preferred in the notion that it harmonizes better with physiological discussions on the cerebral functions, than does the commoner equivalent *Psychical*, which is rather suggestive of the spiritual than of the organic relationships of Mind. In the avoidance of the more ordinary term, however, any sympathy with materialistic views, as such, has to be repudiated; and but a philosophic liberty of scientific speculation claimed as not incompatible with simplicity and faithfulness in matters of belief. The adjective Phrenic has been used hitherto in physiology as denominating a respiratory nerve going to the diaphragm, and applied originally on the supposition that that organ—the midriff, which upholds the heart itself, and is so subservient in many an emotion—was no less than the seat of the Mind. It is more legitimately applicable to cerebral functions; and, indeed, as suggested by Dr. Guislain, the term Phrenology would be the best term for the science of the mental functions of the nervous system, could it be successfully reclaimed from the spurious philosophy to which it is allied—as it certainly would have best comprehended technically the observations now offered in elucidation of cerebral action.

Avocations not averse entirely to such speculations have, however, been such as tended to make them be approached quite as often from a medical as from a metaphysical starting-point, rendering an apologetic prologue necessary for any inclination to impose pathological for physiological illustrations. Nor can it be less appropriate to claim, beforehand, some indulgence for bringing forward views which may seem neither very profound nor altogether new in an assembly numbering many acquainted with, and some eminent in, the departments of physiology and psychology.

The observation that the Mind is capable of becoming diseased, would indicate either its non-spirituality, its composite constitution, or that it is known to us but by machinery doomed to derangement and decay—for the only psychical disorders are perplexities and errors, or rather there is no spiritual disease but sin. Yet, there is a phrenical pathology; and its sources are two—being material decay on the one hand, or vice on the other—either of which may occasion disturbance of that relationship of spirit and matter by which manifestation of mind is possible.

The study of the human mind may be carried on in several ways—as by subjective questionings of consciousness, by observation of the actions of men, and by examination of the human organism. Metaphysicians have made most of the first way, and physiologists of the last; but a right philosophy determines each mode to be perfect, and requiring to be supplemented by the others.

In self-regarding our minds, we distinguish a set of ideas, and a capacity of regarding these. On continued introspection, it is further seen that the ideas, though a multitude, are really but a succession of individuals coming into consciousness in single file, one by one, and capable of being marshalled by the faculty which is regarding them. That faculty is the Will; and its power over the current of thoughts consists, not in creating them, but in regulating their succession, so that particular ideas may be advanced, detained, superseded, recalled, or otherwise dealt with in the wish of the individual. The Will has a power over the succession of ideas; and when ideas move continuously independently of the Will, the mind is diseased.

But the Will itself is not incapable of being influenced by the thoughts which it is its business to guide. It stirs through the reaction of certain of these upon it—which thoughts are called motives. In a healthy state the Will is free to act or not although urged by motives. It cannot act without a motive; but it need not speak the words whispered into its ear, nor use the weapon put into its hand. It has a choice of action, and a choice of motive; and when it ceases to have, the Mind is diseased.

In the natural Human Mind, then, the Will has a government of the current of thoughts, which, again, seek to move, but do not coerce the Will. Individuality is in the Will; personality is the Will; the Will is the Man. In the same state, the Will is free, and thought and action are neither will-ful, nor will-less; in the insane, the Will is driven by thought, and is blinded, fettered, and overridden by disease.

But if insanity depended in any case on abeyance of judgment, how is it a diseased condition of the volition; and what has disordered Understanding to do with a morbid quality of Will? Will and Understanding have been kept far enough apart on psychologic pages undoubtedly; but truly, does it not seem that not only is there no doing, but that there is even no thinking without the activity of the personal Will? In every action of the mind, thought, feeling, and volition are concerned; for, indeed, consciousness reports itself a unity, and not a muster of related forces. Disordered intellect is disordered mind, and, in that, diseased Will. The Will is the essence of the faculty of attention, which is at the base of all intellectual operations, and that of judgment amongst them. An insane idea has its origin in a loss of healthy action in the mental faculties, and is established and continued through defect in the Will, just as much as a morbid propensity is. Abnormal excitement, depression, apathy, stupidity, folly, and delusion, are phenomena of defective control of thought or action. They are all traceable to a fading personality. The individualism of the subject is less apparent—its limitation less distinct—the ego less asserted—the person is less himself—less a king over his thoughts, which are more and more impressible by external causes, and less and less the reagents of the personal mind. Thoughts possess the man; his organisms are becoming the playthings of sensational influences, so that instead of possessing his ideas, he is, as Coleridge said, possessed by them.

The ideas which furnish the mind, and are in its keeping, have been classified as Ideas of Sensation and Ideas of Reflection.

The former proceed from the action of the material external world on the personality, and the latter from the action of the personality on the Ideas of Sensation. Ideas of Sensation spring from two sources,—the Human Body and the extraneous body of the Universe. They rush inwards towards the conscious Brain, through "the five gates of knowledge"—the Eye, the Ear, and other organs of sense; and rise up also, to the sensorium, from the penetralia of the body itself. Perhaps ideas, that can scarcely be called sensational, may be born with the Brain itself, or descend to it from the universal Pneuma by which it is overarched;* but to speculate so were less metaphysical than mystic, and more imaginative than either. Without adopting such fancies, probably we should take too narrow a gauge of our Ideas, in limiting their origin to the impressions on the nerves of the senses, for there is the certainty of many of our feelings being either morbid or healthy, in harmony with states of welfare, or the reverse, in the hidden chambers of the animal economy, where there is neither eye, nor ear, nor any accredited ambassador from the distant seat of government, and where even a muffled sensibility is very doubtfully recognised. Save as general feelings of comfort, cheerfulness, and self-assuredness, it would be difficult to condescend on any detail of such ideas, under ordinary corporeal conditions; but while the psychologist might scruple to declare them, the observer of the mental phenomena of disease has no hesitation in pointing to the trepidation of the palpitating heart, the moroseness of the disturbed liver, and the poltroonery or hysteric mobility of lowered sexual vitality, as evidentiary of the field of sensation that may lie within the barriers where the external senses are placed. Nay, some speculations have not rested at the organs of the body, but have wished to establish the recognition of a communion, through the living blood, of all the conservative fluids of the economy with the humanizing influences of the Brain.

The external world, both of foreign material objects and of the somatic phases of the individual, acting brainwards, constitutes but the receptivity or passive voice of Mind. There is an ability of reacting on the external world; and in the mind there are ideas of Reflection, as well as of Sensation. It possesses a power that rules the ranks of ideas as they rise, or even recalls them from the limbo of thought into which they sink, so that they move again like ghosts of themselves across the cerebral tracks, or start up in a resurrection transcending all sensational experiences. It is not our object, however, at present, to demonstrate the reflective capacities and activities of mind, and to dwell on such phenomena as Memory and Imagination abstractedly from their

* Gardiner Wilkinson.

organic relationships, and, therefore, let us hurry forward to the consideration of the corporeal machinery connected with phrenic action, and the physiological rather than the metaphysical view of the subject.

For the production of the Human Mind, phenomena are observable pointing to a union of the spiritual with the material, in the way of sympathetic, parallel, synchronous, or, as is most likely, unnameable and inscrutable relationship and communion. In truth, we do not know Mind, but only Humanity. A mere consciousness of ideas of sensation and reflection could never have found its way into a nosology, or into an hospital, or been treated or mistreated as efficiently by a Doctor in Medicine, as by a Doctor in Philosophy. Mind has a recognition in practice of Physic as well as in impractical Metaphysics. The only mind we know at all, and can seek to investigate, is the Human Mind; and it is self-asserting and individual in others, quite as much as it is conscious in ourselves. There is a faculty of Will, which in us compels acknowledgment of itself by our fellows, and in them a recognition by us. I know my own faculties subjectively, but those of others only sensationally. Of me, none but I can have any knowledge, save of those things which my body does. Subjective knowledge has an incompleteness in it practically, and mere observation is lacking in profundity. We know not Mind but Humanity, and that inefficiently. We know nothing of each other but the telegraphing of our muscular systems, and the indicia of their working, as recorded on matter around us. *Ego* and *non-ego* are not self-limited and apart more than are *ego* and *tu* separated from absolute observation of each other by a hedge of muscular movement. Sensation, perceived in others, is but motion perceived; will, but directed movement; and emotion, but physiognomical expression. For example: What do we know of anger, but that it is a peculiar action of the facial muscles, with, probably, associated violence and malevolence of deed; of fear, but that it is tremor, flight, paralysis, and so forth; of talent, but that it is the successful result of endeavour; or of genius, but the exhibition of new power? So, to harangue from the pathologist's pulpit, in relation to disease of mind, lunacy is simply morbid action, insanity nothing more specially mysterious than sorrowing vitality is mysterious—only a sick man making an unhappy self-assertion of absurdity, and requiring the attention of the physician and pharmacy but to reform his gymnastical deportment, so that he shall, peradventure, once more appear to be, and be denominated wise. Medico-legally, as you may be aware, it does not signify at all what hallucinations a man has, but only what delusions he believes in and obeys to do folly; and, therefore, that it is the doer of foolish things that is lunatic, and only the madman that is insane. The Fatuus and Furiosus, only they, are the freemen of the asylum, and the freedmen of the social world.

From such views it may be stated, that a man's Mind is a movement betwixt feeling and action—a spiritual intervention betwixt organs of sense affected by impressions of matter on the one hand, and matter affected by muscular action on the other. Conversely, Body is a substantial nexus of feeling and willing.

All phrenical action, from the lowest manifestations in the animal world to the highest self-consciousness in man, is educed from animal machinery by the traction of a loving Spirit which no human science has apprehended.

We seek but to understand something of the phreno-corporeal relation from the corporeal side.

So far as physiology at present guides us, it would seem that the manifestation of mind in the human body, and indeed phrenic action under every circumstance of life, is dependent on the intervention of the grey nervous cell between spiritual influence and perceptible matter. To affect thought, matter must act through this channel; and to influence matter, mind must start forward from the same intermedium. In phrenic action these cells wax and decay, and are renewed, by an unseen stimulus acting on a living organism, whose vital capacity of so responding has been thought to be associated, in a more special degree, with the phosphoric element of nervous tissue, than with any other of its chemical constituents.

In *Sensibility*, which is the base of all phrenic manifestation, and the root whereby the blossom of mind is nourished from the soil, the grey nervous tissue, which, in the form of a cell-nucleus, terminates every sensitive nerve, is impressed by the contact of an external object. This impression is conveyed through a susceptible track of white nervous matter, called a nerve, to a mass of grey neurine—a mass of nervous cells—called a ganglion, where the inward moving impression loses its character, and is reflected outwards as a reacting force. This force is conveyed from the ganglion also by a nerve, which, however, does not end in grey neurine, but in muscular fibre. The muscle, thus quickened, indicates the establishment of sensibility by contracting, and thus more or less directly reacting on the external matter, whose impression was the beginning of this chain of vital action.

Such is the apparatus of sensibility; though not seen so disguised in simplicity, but always elaborated; the cell-nucleus and its adjuncts being surrounded by structures framing organs of sense, and the opposed contractile fibres being made up with fulcra and levers into animal forms. Sensibility need have no connexion with Mind. It is not mind; but it always underlies it. It is sensation without consciousness; and is, simply, what is known to physiologists as irritability, nerve-action, spinal or reflex action, a phenomenon in living bodies which is not phrenic, and for which no cerebrum is required.

M. Matter. g. Termination of sensitive nerve: organs of sense. N. Nerve: white nervous tissue. G. Ganglion: grey nervous tissue. C. Muscle. S. Instrument of reaction: skeleton, &c.

The above is a representation of vital reaction, not of sensation. Sensitive nerves are, it is true, employed; but before they can be the means of conveying conscious sensation, they must form part of a more complex nervous arrangement. The apparatus must be enlarged in the ascending direction by addition of grey nerve-tissue; so that when the sensitive nerve is affected by external stimulation, the impression conveyed along the nerve, after reaching the before-mentioned ganglion, instead of being, as formerly, immediately indicated outwardly by reflex action, travels upwards to a second ganglion, where the impression becomes a *Perception* or conscious sensation, and its external manifestation in the muscular system an expression of *Emotion*, and an indication of pleasure or pain.

The following diagram represents the machinery required for the commencement of phrenic action, and the establishment and indication of consciousness. Mind was not indicated before this development was given; consciousness was not born, and no cerebrum was required. As yet this child is but infantine; and the phrenic capacity represented demands but a small brain, made up but of the Thalami Optici, Corpora Olivaria, and sensorial ganglia, and of the Corpora Striata, and motor ganglia.

Emotional acts, though the outward indication of sensation, do not express merely the perceptions derived from the five external senses, but also those less fully apprehensible which rise out of the common sensation of the body—such as hunger, thirst, fatigue, and many other feelings which are peculiarly liable to be morbidly expressed. When it is well with organs, tissues, and fluids, in the hidden chambers of the body, there will be a declared manifestation of cheerfulness, activity, and natural affections; when otherwise, more probably melancholy, apathy, and deranged impulses. Not that such conditions are in every case so generated, even when they are the undoubted offspring of bodily disease, for their origin may be at the nervous centres as well as at the nervous extremities.

Before going on further with this synthetic exposition of phrenic action, and the demonstration in a diagrammatic way of its machinery, it has to be stated, what may not be indicated by signs or symbols, that the mind is more than a recipient and exponent of sensational impressions and emotional intercourses. While the stimuli which pass inwards as sensations are transmitted outwards as muscular force, they have left something behind, which has been called *experience,* in the charge of a certain capacity of retention in the nerve tissues, named *memory,* and which is reproducible in *recollection.* In what way soever sensations are conveyed onwards from the terminations of nerves through nervous track to nervous track, from ganglion to ganglion—whether by vibration, current, or in whatever unknown way—this current, or its effects in the nervous tissues, would seem to be reproducible therein—not merely by the renewal of the external stimulus, but also by an internal excitation passing downwards to the seat of sensorial perception from regions higher than those to which we have yet inquiringly ascended. The sensation is again felt, and, perhaps, even its physical indications manifested by its corporeal machinery. I repeat: the ganglia of perception and emotion are capable of being influenced by a recurrent stimulation generated internally, and transmitted to them from parts higher up in the nervous scale, and which causes them to reproduce and re-act whatever impulses had come to them from without. The ganglia of perception have a valuable treasure in them in this way, but they have no mine wider or profounder than their experience, however importunate may be the craving of imagination.

The human brain being more than a mere sensorium, is not culminated or domed by the ganglia of perception; and conscious sensation is not therefore manifested outwardly in expressed emotion as the necessary sum and completion of phrenic phenomena, but travels upwards, from the domain of perception, to ganglia which are superimposed in position, and thereby dominant in authority. The impression, which had become a part of consciousness in perception, passes along a nervous track to the ganglia which form the seat of the *Will;* and when the force is at last diverted externally to the muscular system, it is now made apparent, not in the guise of instinctive expression of pleasure or pain, nor as simple reflex action, but as voluntary motion or volition.

The nervous superaddition now referred to is the proper beginning of the organism related to the operations of the Human Mind. Unless the original stimulus be powerful to produce a vital impression, and the impression distinct enough to be advanced to the seat of sensation, and its perception be successful in stimulating volition, it does not contribute in any degree to the furnishing of the mind. The Will is the gate through which ideas must be ushered to become objects to the personal consciousness, and is the outlet by which go forth all the manifestations of individualism. The fundamental intellectual process is what we term the *Attention*—which is but the potency of the

Will over perception; and the base of all power is the guiding rein by which volition holds muscular contraction, and composes mere spasm and convulsion into resistance and endeavour.

There are no mental powers, but only a mental power—viz., the Will.

Speculatively, it is a power governing the current of thoughts, and evolving from their movements and associations the various operations of retaining, recalling, combining, alternating, and so forth, exhibited in recollection, fancy, judgment, and other so-called *faculties* of the mind; while, practically, it gives a character of emancipation to forces which without it were fixed in mechanical-like formality both of direction and degree, instead of being outwardly exhibited, variously modified by the fiat of the free internal Will, or even being entirely suppressed within the consciousness of the man himself, were such latency the dictate of an advised and instructed volition.

The seat of the Will is in the hemispherical ganglia of the brain.

The nervous impression, which we have traced as an assumed current from point to point, as becoming successively sensibility, sensation, and volition, and as exhibited in reflex muscular action, emotive expression, and voluntary motion, may rise yet higher than the region of the Will, and have more transcendent and excellent manifestation carried onwards, by reference of the Will, through the channels of *reflection*, to the stage of nervous organization and development, where mere reflex movement—which had first become conscious, and next voluntary—may yet become rationally controlled before outward declaration.

Ascent carries forward the nervous current, in the act of reflexion, through the ganglion of *Reason*, to be displayed externally as voluntary muscular motion rationally controlled, or it may be rationally suppressed. The establishment of an authority higher than volition leads us to a brief consideration of the relation of humanity to freedom and fatalism. Humanity is the manifestation of the Will of Man. The will acts from *motives*, and cannot act but by the influence of motives. These are often denominated *lower* or *higher*. What do these terms mean in the physiology of phrenical action ? They mean, that when motives are such as to be called lower, they are ideas coming from ganglia lower than the seat of volition in the phrenic organism ; and that when they can be called higher, they are the judgments of Reason, or the controlling influence of ganglia elevated above that of the Will. The lower motives are independent by their birth, and tyrannous and unrestrained by inclination. They have a guaranteed access to the Will, but the Will need not obey them. They are the subject mob over which the Will is king. It is the Will that says amen to desire; instead, however, of transmitting its tumultuous urgency onwards to a termination in deeds—instead of converting sensational impulses into motor force—it may direct the current upwards, so to speak, into the sphere of Reason, and first regulate its expression by control. The higher motives are not so chartered; they do not urge the will unsought, as do the lower motives, but come only when called for by the Will, and act only when thus permitted. The degree in which the Will is subjected to the higher motives is dependent on the phrenical habits of the man himself. His Will is free. It may obey the lower motives, but it may refer them to the judgment of the Reason and disobey them, preferring the approval of this and such authority to the gratification of sense or desire. Necessity compels to action, but does not govern the deed.

The Will is the fulcrum of all muscular action that is phrenically instituted. All subordinate points of vitality in cerebral function—all sensibilities—all their corporeal expression—form

SPIRIT.
FAITH.
LOVE.
REFLECTION.
REASON.
CONTROL.
ATTENTION.
WILL.
VOL. MOTION.
SENSATION.
PERCEPTION
EMOTIVE EXPRESSION.
EXT. IMPRESSION.
VITAL ACTION.
REFLEX ACTION.
MATTER.

but footsteps of the mental throne on which the volition is installed as king; and aught higher, as the Reason, whether reflective or intuitional, is but an oracle which the king cultivates and consults, just so far as he chooses to seek piety and wisdom.

Here, in the hemispheric ganglia of the brain, is the terminus of the nervous chain, and the completion of the animal machinery or corporeal apparatus of mental action: not that we are to believe that on this mountain we have but to sit down, and either look back to the clods in the valley from which we have ascended, or ruminate in self-absorbed regard on the fortunate adaptations for enjoyment to be observed in the body, and the wonders of which carbon and water are capable in a human frame; no! for we leave the intelligence in the act of aspiring; and although here, where anatomical investigation becomes amaurotic, and the physiologist has to be dumb, our diagram must terminate, we believe that from this Jacob's ladder the Reason takes wings to itself, and soars upwards by *Faith* to a Spirit acting downwards towards humanity in *Love*.

The steps through which we trace the growth of the human mind are—from organic movement to consciousness, from consciousness to volition, from volition to reason, and from reason to the capacity of acknowledging a guiding power beyond the limits of organic life. We have looked upon phrenic effort as commencing in mere reflex action, and carried upwards successively through instinctive manifestation, voluntary motion, and rational action, to the development of a will capable of desiring to act in unison with a holier will.

Humanity is the highest phasis of the relation of Spirit to Matter in this world, and is the Human Mind for all philosophic apprehension of psychology. At the base is a vitality which is simply uterine; above that, merely animal; farther forward, only foolish; above this, diabolic; and, only when transcending mere intellectual power, graduating at Humanity, and becoming capable of a life which may blossom as Christian in this world, and sow the seeds of a development that will yet be more godlike, and of a life that will be immortal in heaven.

## Figures and Tables

**Figure f1:**
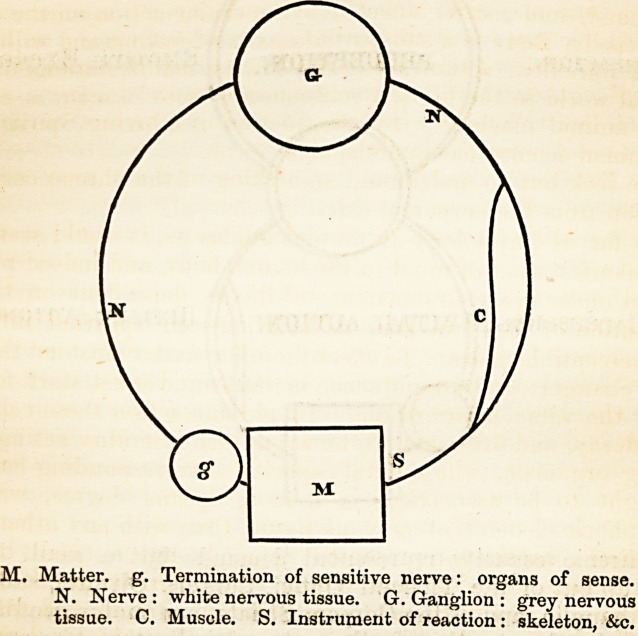


**Figure f2:**
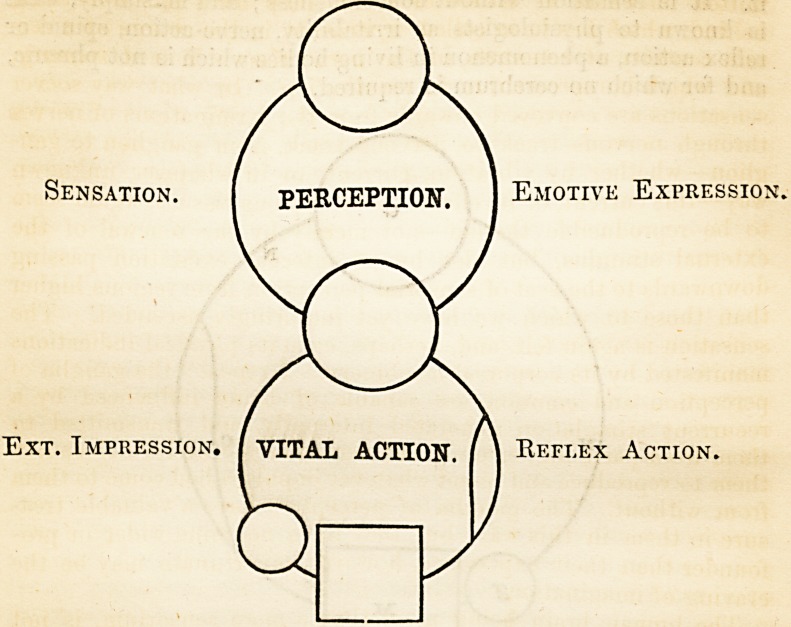


**Figure f3:**
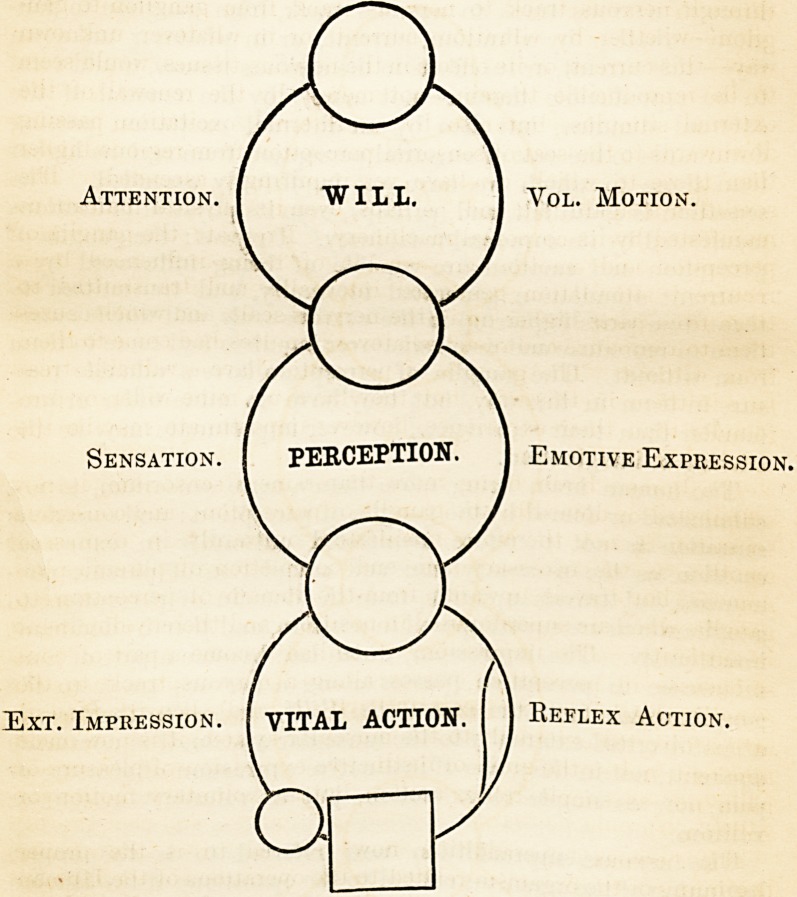


**Figure f4:**
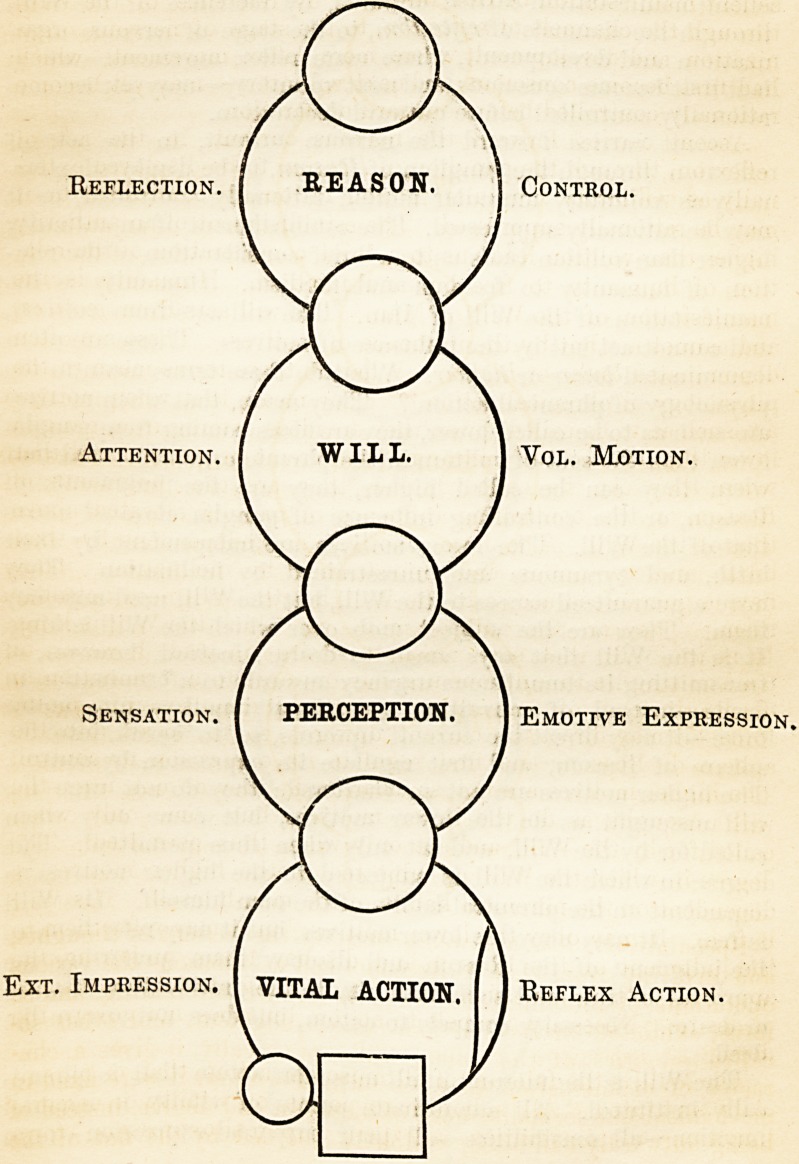


**Figure f5:**